# Evaluation of a Loop-Mediated Isothermal Amplification Assay to Detect Carbapenemases Directly From Bronchoalveolar Lavage Fluid Spiked With *Acinetobacter* spp.

**DOI:** 10.3389/fmicb.2020.597684

**Published:** 2021-01-13

**Authors:** Javier Moreno-Morales, Andrea Vergara, Tomislav Kostyanev, Jesús Rodriguez-Baño, Herman Goossens, Jordi Vila

**Affiliations:** ^1^Institute for Global Health (ISGlobal), Hospital Clínic – Universitat de Barcelona, Barcelona, Spain; ^2^Department of Clinical Microbiology – CDB, Hospital Clínic, University of Barcelona, Barcelona, Spain; ^3^Department of Medical Microbiology, Vaccine and Infectious Disease Institute, University of Antwerp, Antwerp, Belgium; ^4^Unidad Clínica de Enfermedades Infecciosas, Microbiología y Medicina Preventiva, Hospital Universitario Virgen Macarena/Departamento de Medicina, Universidad de Sevilla/Instituto de Biomedicina de Sevilla (IBiS), Seville, Spain; ^5^Laboratory of Medical Microbiology, University Hospital Antwerp, Antwerp, Belgium

**Keywords:** carbapenemases, *Acinetobacter* spp., bronchoalveolar lavage, detection, oxacillinases

## Abstract

Carbapenem-resistant *Acinetobacter* spp. mainly *Acinetobacter baumannii* are frequently causing nosocomial infections with high mortality. In this study, the efficacy of the Eazyplex^®^ SuperBug Complete A system, based on loop-mediated isothermal amplification (LAMP), to detect the presence of carbapenemases in *Acinetobacter* spp. directly from bronchoalveolar lavage (BAL) samples was assessed. A total of 22 *Acinetobacter* spp. strains producing OXA-23, OXA-40, OXA-58, NDM, and IMP were selected. Eazyplex SuperBug Complete A kit, used with the Genie II device, is a molecular diagnostics kit that detects a selection of genes that express carbapenemases (*bla*_*KPC*_, *bla*_*NDM*_, *bla*_*VIM*_, *bla*_*OXA–48*_, *bla*_*OXA–23*_, *bla*_*OXA–40*_, and *bla*_*OXA–58*_). Negative BAL samples were identified, McFarland solutions were prepared from each of the 22 *Acinetobacter* strains and serial dilutions in saline solution were made to finally spike BAL samples to a concentration of 10^2^ and 10^3^ CFU/ml. Fifteen concentrations out of the 44 tested out did not provide detection of the carbapenemase-producing gene, all but one being at the lowest concentration tested at 10^2^ CFU/ml; therefore, the limit of sensitivity is 10^3^ CFU/ml. This assay represents the kind of advantages that investing in molecular diagnostics brings to the clinical practice, allowing the identification of carbapenemases in less than 30 min with a sensitivity of 10^3^ CFU/ml.

## Introduction

Carbapenems are potent β-lactam antibiotics with broad-spectrum and bactericidal mode of action (Codjoe and Donkor, 2017). Their use was increased due to the spread of extended spectrum β-lactamase-producing *Enterobacteriales* toward whom they are active (Hawkey and Livermore, 2012; Bush and Bradford, 2020). Carbapenems are considered one of the most efficacious antimicrobials to treat bacterial infections (Codjoe and Donkor, 2017). However, resistance by carbapenemases did not take long to appear, and it poses a major threat to public health (Hawkey and Livermore, 2012).

*Acinetobacter* spp. members and specially carbapenem-resistant *Acinetobacter baumannii* (CRAB) are among the world’s most dangerous pathogen threats. CRAB has been classified as a critical priority pathogen by the WHO’s priority pathogens to guide research and development (R&D) of new antibiotics (Tacconelli et al., 2018; World Health Organization, 2019) and as an urgent threat that requires aggressive action by Centers for Disease Control and Prevention (CDC) (Centers for Disease Control, 2019).

Even though community-acquired *Acinetobacter* infections can occur, the most common and acute infections happen in the nosocomial setting. *Acinetobacter* lurks around intensive care units and surgical wards causing a number of infections (e.g., on burns and soft tissue, urinary tract, and bloodstream) and specially ventilator-associated pneumonia (VAP) in patients under mechanical ventilation (Evans et al., 2012; Bush and Bradford, 2020).

Ventilator-associated pneumonia develops in intensive care units in patients under ventilation for at least 48 h. Rapid diagnostic of VAP-causing pathogens is of utmost importance: VAP patients not only have longer hospital stays and need more antibiotics, therefore their treatment is more expensive, but also have higher mortality (Torres et al., 2017; Bonell et al., 2019).

This study aims to evaluate Eazyplex^®^ SuperBug Complete A based on loop-mediated isothermal amplification (LAMP) for detecting carbapenemase produced by *Acinetobacter* directly from inoculated bronchoalveolar lavage (BAL) samples.

## Materials and Methods

Eazyplex^®^ SuperBug Complete A kit (AmplexDiagnostics GmbH, Germany), used with the Genie II device (OptiGene, Horsham, United Kingdom), is a molecular diagnostics kit that detects a selection of genes that express carbapenemases (including metallo-β-lactamases and oxacillinases). Results after detection of bacterial DNA are presented within 30 min.

The kit is composed of eight tube strips each with a mix of lyophilized agents for the amplification of one of the following seven genes: *bla*_*KPC*_, *bla*_*NDM*_, *bla*_*VIM*_, *bla*_*OXA–48*_, *bla*_*OXA–23*_, *bla*_*OXA–40*_, and *bla*_*OXA–58*_. The eighth tube is an internal inhibitory control.

Once the samples are prepared and the strip is inside the Genie II device, a LAMP is performed. The reaction is incubated at 66°C for 30 min, and detection is performed *via* fluorescence excitation, for up to two strips at a time.

A total of 22 *Acinetobacter* spp. strains producing OXA-23, OXA-40, OXA-58, NDM, and IMP were selected (Table 1). Isolate identification was performed *via* MALDI-TOF/MS (Bruker Daltonics, Bremen, Germany). Carbapenemase gene detection was checked *via* conventional PCR for each of the strains (Woodford et al., 2006; Kulah et al., 2010; Solé et al., 2011). The strains selected basically were epidemiologically unrelated due to its different sequence types (ST) or geographical origins.

**TABLE 1:** Eazyplex SuperBug Complete A kit was used to detect carbapenemases of *Acinetobacter* spp. from spiked BAL.

**Strain**	**Resistance gene (*bla*)**	**Concentration (CFU/ml)**	**Eazyplex SuperBug Complete A (minutes:seconds)**
*A. baumannii* 1	OXA-23	10^3^	14:00
		10^2^	16:15
*A. baumannii* 2	OXA-23	10^3^	11:45
		10^2^	–
*A. baumannii* 3	OXA-23	10^3^	28:00
		10^2^	21:15
*A. baumannii* 4	OXA-23	10^3^	13:00
		10^2^	–
*A. baumannii* 5	OXA-23	10^3^	11:15
		10^2^	14:15
*A. baumannii* 6	OXA-40	10^3^	12:50
		10^2^	13:15
*A. baumannii* 7	OXA-40	10^3^	13:30
		10^2^	–
*A. baumannii* 8	OXA-40	10^3^	11:30
		10^2^	17:00
*A. baumannii* 9	OXA-40	10^3^	9:45
		10^2^	–
*A. baumannii* 10	OXA-40	10^3^	9:30
		10^2^	10:30
*A. baumannii* 11	OXA-58	10^3^	16:10
		10^2^	18:30
*A. baumannii* 12	OXA-58	10^3^	16:15
		10^2^	–
*A. baumannii* 13	OXA-58	10^3^	16:45
		10^2^	21:30
*Acinetobacter nosocomialis* 1 OXA-58 IMP-4*	OXA-58	10^3^	14:30
		10^2^	–
*A. baumannii* 14	OXA-58	10^3^	9:30
		10^2^	10:00
*A. baumannii* 15	OXA-58	10^3^	12:15
		10^2^	12:45
*Acinetobacter pittii* 1	NDM	10^3^	20:00
		10^2^	–
*Acinetobacter dijkshoorniae* 1	NDM	10^3^	18:30
		10^2^	–
*A. baumannii* 16	NDM	10^3^	–
		10^2^	–
*A. baumannii* 17	NDM	10^3^	13:45
		10^2^	–
*A. junii* 1 IMP-1*	IMP	10^3^	–
		10^2^	–
*A. baumannii* 18 IMP-2*	IMP	10^3^	–
		10^2^	–

Eazyplex^®^ SuperBug Complete A detection time per concentration tested for each of the *Acinetobacter* spp. strains tested. “–” indicates no detection of the carbapenemase-producing gene in the concentration tested. *IMP-producing strains were included in the study to cover the range of carbapenemases found in *Acinetobacter* although not being detected in the kit.

Negative BAL samples were identified and collected at the Clinical Microbiology Laboratory from Hospital Clinic of Barcelona; samples were stored at −80°C. McFarland solutions were prepared from each of the 22 *Acinetobacter* strains, and serial dilutions in saline solution were made to finally spike BAL samples to a concentration of 10^2^ and 10^3^ CFU/ml.

The protocol consisted in: centrifugation of 850 μl of the 10^2^ and 10^3^ spiked BAL samples (at 14,000 *g* for 5 min), addition of 500 μl of resuspension and lysis fluid (RALF, provided with the kit) to the pellet obtained, incubation at 99°C for 2 min and a final centrifugation step (4,000 rpm for 2 min). Finally, 25 μl of the supernatant was added to each tube of the assay strip. The hands-on time took a maximum of 15 min per strain.

## Results and Discussion

Increasing resistance to antimicrobials and specifically carbapenems is reported in *A. baumannii* in the past years. In Spain, there has been an increase of up to 40% in *A. baumannii* clinical isolates presenting resistance to carbapenems from 2000 to 2010; 86% of the 446 *A. baumannii* clinical isolates presented resistance to carbapenems in 2010’s study (Fernández-Cuenca et al., 2013).

This situation is rather common. Recently, European Centre for Disease Prevention and Control (ECDC) has reported ≥50% of *Acinetobacter* spp. invasive isolates present resistance to carbapenems in Hungary, Poland, Bulgaria, Latvia, Italy, Spain, Cyprus, Romania, Lithuania, Greece, and Croatia in 2018’s Annual Report of the European Antimicrobial Resistance Surveillance Network (European Centre for Disease Prevention and Control, 2018).

Current effective antibiotics for the treatment of CRAB are scarce and are not the most suitable therapeutic agents due to poor pharmacokinetics, toxicity (as in the case of polymyxins), and emergence of resistance (Garnacho-Montero et al., 2015). Chromosome and/or plasmid encoded carbapenemases are the main mechanism of resistance to carbapenems in CRAB (Roca et al., 2012; Bush and Bradford, 2020); thus, rapid detection of carbapenemases is key to guide effective antibiotic therapies (Garnacho-Montero et al., 2015).

Although the Complete A kit is not specific for *Acinetobacter*, carbapenemase coverage is enough to check for the main carbapenemases present in *Acinetobacter*. Of all the carbapenemases described in *A. baumannii*, oxacillinases are by far the most frequently found among the most prevalent clones, being the international clone 2 OXA-23 producing *A. baumannii* worldwide spread.

Detection of carbapenemase-producing genes in the tested strains using Eazyplex SuperBug Complete A assay is shown in Table 1, and the results agree with conventional PCR results. Detection time values vary per strain and gene. Only 15 concentrations tested out of 44 did not provide detection of the carbapenemase-producing gene, all being at the lowest concentration tested at 10^2^ CFU/ml; therefore, with the limit of sensitivity being 10^3^ CFU/ml, we consider that this kit has enough sensitivity for the detection of carbapenemase-producing *Acinetobacter* in clinical BAL samples, taking into consideration that the cut-off for BAL is 10^4^ CFU/ml.

With a maximum hands-on time of 15 min per sample and 30 min run time (approximately 45 min total), this assay proves to be a great advantage compared to routine methods in the clinical microbiology laboratory that need 16–24 h for results to be obtained. Naturally, further antimicrobial susceptibility testing should be considered in all samples. The only rapid test (4 h) that allows the detection of the most frequent pathogens causing HAP is the Unyvero Hospitalized Pneumonia (HPN; Curetis GmbH, Germany). This multiplex panel also includes some resistant markers (18 in total); among them, it can detect the genes encoding VIM, IMP, NDM, KPC, OXA-23, OXA-24, OXA-48, and OXA-58. However, as far as we know, it has not been validated for detection of carbapenemases in *A. baumannii.*

We visualize the following workflow for diagnosis of hospital-acquired pneumonia (HAP): when the sample arrives to the clinical microbiology laboratory, rapid identification of the bacteria causing HAP is performed also using an in-house LAMP reaction approach (Vergara et al., 2020a); if *A. baumannii* is identified as the pathogen causing the infection, the method to detect specific carbapenemases in *Acinetobacter* described in this study is performed. However, if Enterobacterales is detected, the same approach can be applied (Vergara et al., 2020b).

Using Eazyplex^®^ SuperBug Complete A assay will allow to guide and optimize antibiotic therapies earlier than with usual techniques used in the laboratory, which likely means a decrease in mortality. This assay represents the kind of advantages that investing in molecular diagnostics brings to the clinical practice: allows the identification of specific resistance mechanisms in approximately 45 min and if sample identification using LAMP was included as a first step (1 h), both pathogen and resistance mechanism could be identified in less than 2 h.

## Data Availability Statement

The original contributions presented in the study are included in the article/supplementary material, further inquiries can be directed to the corresponding author/s.

## Author Contributions

JV, JR-B, and HG: conceptualization, design, and writing–review and editing. JM-M and TK: methodology. AV: supervision and validation. JV: writing–original draft preparation and funding acquisition. All authors contributed to the article and approved the submitted version.

## Conflict of Interest

The authors declare that the research was conducted in the absence of any commercial or financial relationships that could be construed as a potential conflict of interest.
